# The class inclusion question: a case study in applying pragmatics to the experimental study of cognition

**DOI:** 10.1186/s40064-016-2467-z

**Published:** 2016-07-19

**Authors:** Guy Politzer

**Affiliations:** Institut Jean Nicod, Ecole normale supérieure, 29 rue d’Ulm, 75005 Paris, France

**Keywords:** Cognitive development, Class inclusion, Categorisation, Privative ambiguity, Relevance theory

## Abstract

For more than 70 years, Piaget’s class-inclusion task (given, e.g., five asters and three tulips, the child is asked whether “there are more asters or more flowers”) has been the object of experimental investigation. Inclusion is of considerable importance for cognitive science as it is a key concept for logical operations and knowledge representation. It is shown that the question can be characterised by a kind of privative ambiguity which is at the source of the younger children’s answer, “more asters”. A relevance-theoretic explanation of children’s interpretation of the question and of the subsequent responses is expounded. This account can explain the effect of all the factors that are known to influence performance (e.g., role of collections, counting, typicality, qualification, syntax, etc.), a review of which is presented. It is further tested experimentally. The development of performance is explained on the basis of the way children disambiguate the question. This study exemplifies the two ways in which pragmatic analysis is pertinent to the study of children’s (as well as adults’) reasoning and judgement, namely in explaining and predicting participants’ comprehension of the statements and questions, and in taking into account attribution processes that occur in the experimental setting.

## Background

Since its first appearance more than 70 years ago (Piaget and Szeminska 1941) the class inclusion question has given rise to countless investigations. Consider the paradigmatic case. A child is presented with the picture of a set of, say, seven flowers comprising two subsets of five asters and two tulips. When young children are asked, “Are there more asters or more flowers” (the standard form of the class inclusion question), the typical answer is that there are more asters. These children are said to fail the class inclusion question. Conventionally, a population is deemed to pass the question when the criterion of 50 % of answers “there are more flowers” is reached. Averaging across studies, this occurs between eight and nine years of age among school children in the western world (Winer 1980). The significance of this result in relation to the existence of many factors that influence performance has been hotly debated, both inside and outside Piagetian theory, with a peak in the 1970s and 1980s.

The concept of inclusion is of considerable importance for cognitive science. The theoretical interest to explain when and how an awareness that the whole is more than one of its parts emerges in children’s cognition is uncontroversial. And how hierarchical categories are learned, represented and exploited is one of the major problems of knowledge representation. The human mind has the remarkable capability to treat a set and one of its subsets simultaneously, that is, treat the extension of a subset while recognising at the same time that its members belong to an including set. At a mature level of development, this manifests itself, in particular, by the ability to consider one given entity as an A or a B (with B included in A) simultaneously. Earlier in development, the child learns that a name can refer both to a basic level and a superordinate level. Using a variety of experimental procedures, many studies have addressed the question of the conditions that foster this ability and of the age at which it emerges. Because there is evidence that this occurs as early as three years of age (Blewitt 1994; Callanan 1989; Diesendruck and Shatz, 2001; Johnson et al. 1997; Mervis et al. 1994; Nguyen and Murphy 2003; Taylor and Gelman 1989; Waxman and Hatch 1992) the fact that success on the class inclusion question appears about 5 years later seems deeply mysterious.

This paper aims to re-examine the class inclusion problem in the light of relevance theory (Sperber and Wilson 1995). It will be organised as follows. To begin with, a fundamental distinction between two psychological levels of attainment of inclusion will be recalled. Then, the pragmatic analysis of the task will be developed and an explanation of the performance will be presented. An interpretation of the influence of the various factors known to affect performance will follow. Next, a number of experiments that test the pragmatic approach will be described, some of which give an explanation for the late success at the task. Finally, two other theoretical approaches will be considered.

## A fundamental distinction

The following notations will be used. We will be concerned with classes, most of the time partitioned into two. One class, called the *superclass* (noted A) includes two classes, B and B’, called the *subclasses*. B, which has the greater extension, is called the *major* subclass and its complementary B’ is called the *minor* subclass. The names used to denote A (e.g., *flower)* on one hand, and B and B’ *(aster, tulip)* on the other hand are the hypernym, and the hyponyms (majority, minority), respectively.

Piaget was interested in the judgement of the *necessity* of the inclusion of a part in the whole, as this is the hallmark of the achievement of a formal structure. For this, two conditions must be met: (i) the whole class must be permanent to conserve its extension when the child considers the subclass, and (ii) the subclass must be characterisable by subtraction, that is, B’ must be understood as the A that are not B (as well as A is the union of B and B’), which defines the organisation of class addition and subtraction within a reversible system. In Piagetian theory, its is assumed that children’s incorrect response to the class inclusion question is due to a comparison of B with its complementary B’. The reason for this comparison is that the quality of the whole class A has been transferred to B’, which in turn is due to the absence of attainment of the reversible system: When the child isolates B by subtraction, the whole class A stops existing (and vice versa, when the child adds B and B’ to constitute the whole, each subset stops existing). Within the Piagetian framework, the class inclusion question is valid internally (by construct) and externally in the sense that the interview methodology enables the experimenters to ascertain their judgement by analysing the child’s justifications and by looking for resistance to counter-suggestions.

Now a different and more simple use of the task can be made. As Smith (1982) has cogently argued, studying whether children answer the class inclusion question correctly may correspond to another research interest, such as, “Are children aware, *on the basis of the observation,* that there is more in the class than in the subclass presented to them”. Indeed, investigators in the Piagetian tradition used to carefully distinguish *empirical* solving and *logical* solving (Bideaud and Lautrey 1983). Outside Piagetian theory this question, which can be called the *simple* judgement of inclusion, has been the focus of most researchers’ interest. This interest is justified because the simple judgement is the one that is relevant to fundamental aspects of knowledge representation such as the acquisition of hierarchical classifications. The simple judgement differs deeply from a judgement of necessity. Structurally, the latter results from a deductive system, which is not the case for the former. Functionally, the essential difference is that the simple judgement requires empirical knowledge whereas the necessity judgement needs no observation. More importantly, the necessity judgement requires of the child a meta-knowledge, that is, the use of a principle that is built upon the knowledge at work in the simple judgement.

This notion seems to be widely accepted. For example Mandler (1983) suggests that “to answer the typical class-inclusion questions may require the ability to reflect on the implications of one’s knowledge” (p. 120). In considering conscious abstraction as one of the conditions of the understanding of the class inclusion question Piaget (1977) explicitly and specifically expressed this view. Neo-Piagetians such as Moshman and Timmons (1982) posit that the development of metacognition is one of the processes at the origin of logical necessity. Moshman (1990) regards metacognitive awareness as the very object of the development of logical reasoning beyond the age of 5 or 6.

Ironically, cognitive psychologists who are interested in the simple judgement of inclusion have adopted the Piagetian class inclusion question, which was designed, and is valid, for the judgement of necessity, without questioning its validity to study the simple judgement. Based on a pragmatic analysis of the task, it will be claimed in the present paper that it is invalid. A modified question that is valid will be proposed, from which a different, more correct, developmental pattern of performance for the simple judgement will follow.

## The pragmatic analysis

In this section, we will perform a detailed analysis of the task, which includes a linguistic analysis of the question, and an examination of the peculiarities of the relationship between the experimenter and the child in the experimental setting, following an approach detailed in Politzer (1993) and summarised in Politzer (2004) based on relevance theory.

### Referential ambiguity and the inclusive versus exclusive comparisons

A classic riddle among school children is “What barks but is not a dog?” Solving it at once may be hard even for adults, for reasons that are cognitively interesting. Readers who have failed to find the answer, “a bitch”, probably feel both that they should have been able to answer and that they have been cheated–indeed both intuitions are correct. The pair *dog* (generic)—*bitch* completed with *dog* (male) is of course an instance of lexical markedness, *dog* providing an instance of autohyponymy. That lexemes such as *dog* can refer to the class of all the dogs *(dog*_*0*_*)* or to the included class of male dogs *(dog*_*1*_*)* is exploited in the riddle and sometimes referred to as *privative ambiguity*. What barks (a class that uniquely characterises dog_0_) and is not a dog_0_ belongs to an empty class (barking while not being a dog_0_ is contradictory), hence the failure to find a solution. What barks (dog_0_) and is not a dog_1_ defines the class-solution, *bitch*. If initially you have the interpretation dog_1_, you get it right immediately and there is no riddle proper. Assuming that initially you have the interpretation dog_0_, you get it right or not depending on whether or not you shift your interpretation from dog_0_ to dog_1_. Of course, one could imagine a reverse riddle, in which the initial interpretation is dog_1_ but the required construal to get the solution is dog_0_: “Are there more bitches or more dogs” would be one such question where in the vicinity of *bitch*, dog_1_ is a more likely interpretation than dog_0_. We will say that a comparison of *bitch* with dog_1_ is exclusive (or contrastive) and a comparison of *bitch* with dog_0_ is inclusive.

Now consider the class inclusion question, “Are there more asters or more flowers”. The subclass to superclass structure is identical but there is a lexical difference: Whereas the complementary class dog_1_ is not lexicalised, in the class inclusion question the complementary class is lexicalised (the tulips). However, using a hypernym (in the plural) to refer indifferently to a superclass or to one of its subclasses is always correct literally and generally appropriate in ordinary speech (the exceptions stem from a possible distance between the subclass and the basic level: Microbes generally are not felicitously called *animals*). The hypernym is potentially ambiguous and it is the context of the utterance that guides the interpretation. Coming back to the standard class inclusion question, “Are there more asters or more flowers” it is clear that depending on the context, it may be relevant to give it either interpretation: (i) “Are there more asters or more tulips (flower_1_)” or (ii) “Are there more asters or more flowers_0_.” In the first case the children make an exclusive comparison and answer that there are more asters, the response that is deemed to be incorrect; in the second case they make an inclusive comparison and answer that there are more flowers, for which they are credited with a correct response.

In sum, the foregoing analysis reveals the nature of the class inclusion question, namely a riddle in an experimental setting. There is something queer in the question which would not be used naturally. A speaker would normally exploit one of the various linguistic devices available to disambiguate the question. To invite an exclusive construal one would use the name of the class B’, or if it is unknown or not lexicalised use a qualifier or a deictic marker. To invite an inclusive construal one would use the quantifier *all* (which would oblige one to use a different sentence: “What is more, the asters or all the flowers?”).

Interestingly, the triangular structure is often exploited for rhetorical reasons, in particular in advertisements. A commercial slogan (popular in the 1980s) such as “Don’t buy a car, buy a Saab” has a real impact. Here the sentence initially understood as “Don’t buy a car_0_… ” yields a contradiction as a Saab is a car_0_. Reinterpreted as “Don’t buy a car_1_…” (in which car_1_ is the class of cars that are not Saab), it gets the exclusive interpretation. The cognitive effort is worthwhile in terms of effect as the hearer ends up with “buy a Saab” and “don’t buy a car that is not a Saab”.

We now turn to the determination of the relevance of the question, which depends on the interaction between the child and the experimenter.

### Determining the relevance of the question

Hayes (1972) remarked that the way the class inclusion question is interpreted constitutes a developmental variable. This is a fundamental insight. Once this view is adopted, the disambiguation of the question must be envisaged in relation to the child’s development. From the notion that the children attempt to render the question optimally relevant it follows that the way they do so will vary with their cognitive development. In other words, the interpretation chosen by the children is constrained by their level of development. Therefore, the interpretation can be predicted on the basis of what is likely to be the children’s estimation of the relevance of the question.

To specify what it means for the question to be relevant, we need to analyse the relationship between the child and the experimenter. A question is relevant when it can give rise to an answer that is relevant for the questioner, that is, the answer should satisfy the expectation of relevance attributed by the questionee to the questioner^1^. However, experimental settings have (in common with instructional settings) a specific feature characteristic of the testing situations: It was noted long ago (Searle 1969 p. 66) that the question is a higher order question. In testing situations, when a question of the type “Is it the case that S?” is asked, the answer “yes” or “no” is irrelevant to the questioner; what is relevant is to know whether the questionee knows whether it the case that S, which the questionee is aware of. This applies to school age children who are exposed to this kind of questioning through repeated interaction with teachers. In the frame of an experiment, which generally takes place at school, participants cannot fail to identify that the question belongs to this conventional *genre*.

Now the identification of the kind of knowledge which the child expects the experimenter to wish him or her to exhibit is necessarily bounded by two kinds of limit, which are the child’s own knowledge, and as crucially the child’s meta-knowledge. Obviously the children cannot attribute to the experimenter an interest in knowledge that they do not possess themselves; and not any more to knowledge that they possess but are not aware of. This implies that the children attribute an interest in what they feel is a difficult acquisition (often some skill or piece of knowledge being currently learned), that represents a respectable achievement worthy of consideration. Consider now the younger children who are requested to make a quantitative comparison (more B or more A?). The capability they wish to demonstrate, and in which they will attribute an interest to the experimenter, is that of counting and making additions. This is a fundamental school acquisition, highly valorised. They can achieve this demonstration by making either comparison, but are these equally likely to be chosen?

Consider first the younger children, typically five to seven years old, who are in a situation where they have the choice between an exclusive and an inclusive comparison. There are two main differences between these two possibilities. One, the exclusive comparison is numerically easier as it requires to compare the number of asters with the number of tulips (B and B’) whereas the inclusive comparison requires to compare the number of asters with the number of asters + the number of tulips (B and B + B’). Second, the inclusive comparison does not match the child’s experience (nor the adult’s for that matter) as it hardly has any ecological validity. Indeed comparisons in daily life concern exclusive or, less typically, overlapping classes, and hardly ever included classes. These two differences concur to give the exclusive comparison a definite advantage: It is easier. Because it enables the child to achieve the same result for the least effort, the exclusive comparison has the greatest chance of being chosen.

Consider next the older children, typically 8 years old and above. The elementary arithmetic skills are already an objective of the past (even in case they are not actually attained); their mastery cannot constitute an achievement worth demonstrating to the experimenter. But what is currently emerging is metacognition in the linguistic domain (Gombert 1992) and the logical domain (Moshman 1990; in particular, logical necessity: Cormier and Dagenais 1983; Miller et al. 2000). Significantly, it is from about 8 years onwards that children start to understand riddles based on semantic ambiguity (Bernstein 1986; Kilcher 1991; Shultz 1974; Shultz and Horibe 1974; Sutton-Smith 1976) and about the same age that they start to offer a majority of metalinguistic explanations in response to requests to explain the use of linguistic items (Karmiloff-Smith 1986). The contemporaneous character of the emergence of metacognition (logical and linguistic, including awareness of semantic ambiguity) on one hand, and success on the class inclusion question on the other hand is no coincidence: The former is a condition for the latter.

Essentially, when the logical concept of inclusion has been acquired, this provides the kind of knowledge that the child assumes to be of interest to the experimenter and worth showing her. There is an additional piece of knowledge that the child may wish to exhibit, namely that the hypernym is ambiguous and that it is better to disambiguate by referring to the superclass rather than to the minor subclass B’ because if the experimenter wished to refer to B’ she would have used its name.^2^ In brief, of the two comparisons, the inclusive one now is by far the more relevant. One may add another possible reason to use the inclusive comparison, which concerns children at an intermediate level of development. They might make this choice for exactly the opposite reasons why the younger ones who wished to demonstrate their arithmetic skills opted for the exclusive interpretation. This time, opting for the inclusive interpretation amounts to making the most difficult calculation, but the increase in effort is offset by an important increase in effect (precisely showing their capability of executing the most difficult calculation).

To summarise: depending on their metacognitive development the children can disambiguate the class inclusion question in two ways. The younger make the question relevant by interpreting it as a request for an exclusive comparison of the subclasses; the older, by interpreting it as request for an inclusive comparison of a subclass and the superclass. The choice is constrained both by the cognitive and metacognitive capabilities in the logical or linguistic domains. The standard class inclusion question cannot be a valid test of the simple inclusion judgement because the child is not given a fair opportunity to compare a subclass with the superclass, so that failure does not demonstrate that the child does not possess the knowledge that the part is included in the whole. (On the contrary, the standard class inclusion question, provided it is supplemented with justification, may be a valid test of the knowledge of the necessity that the part is included in the whole, as failure is incompatible with the attainment of the required metacognitive knowledge).

## The factors that affect performance

In this section the various factors known to affect performance on the class inclusion question will be reviewed, and it will be shown that in all cases their effect can be explained by the hypothesis of the referential ambiguity of the hypernym.

### Classes versus collections

The most powerful of the factors that affect performance is the replacement of classes by collections. Markman (1973) used materials such as six dogs (four small, two big) and compared two questions: The class question, “Who would have more pets, someone who owned the baby dogs or someone who owned the *dogs*?” and the collection question in which the collection name replaced the final occurrence of the hypernym: “Who […..] who owned the *family”*. The author reports that more than 50 % of 7-year-olds passed the collection question whereas none of them passed the class question. The study was motivated by the observation that it is permissible to designate the subclass by the hypernym, so that if the children are set to make subclass comparisons (for cognitive, linguistic or perceptive reasons), they may be encouraged to misinterpret the question, which cannot occur with collections as it is not possible to designate the subclass by the word “family”. Surprisingly, even though the ambiguity is well noted, its explanatory role is amalgamated with another factor: This is the notion that in a collection such as a family the subparts (parents and children) stand in a specific relation to one another, which could help apprehending the part and the whole simultaneously. This second explanation will be considered in detail and refuted later (“Experimental investigation of the role of collections” section).

### Lexical definition

Applying a modified version of a procedure initially used by Smedslund (1964) in a battery of Piagetian tests, Carpendale et al. (1996) asked an exclusive comparison (“more horses or more cows”) as a preliminary question that preceded the class inclusion question proper (“more horses or more animals”). This resulted in a substantial increase in performance which can be explained by disambiguation. Because the initial formulation conveyed a request for an exclusive comparison, the subsequent formulation by contrast was unlikely to be interpreted again as an exclusive request—there must be some relevance in the change in wording—so that the inclusive interpretation was chosen by the children who could remember the first request. In brief, this procedure indirectly attracts the attention to the difference between naming the minority hyponym and the hypernym. It is remarkable that Carpendale et al. (1996) discuss at some length the pragmatic explanation, but in the end reject it on the grounds that they do not see how to account for the developmental trend in the performance on the class inclusion question.

Winer and Falkner (1984) showed a dog to two groups of adults. The first group was asked, “Is it a dog or an animal?” and the second, “Is it a dog or an animal, or both?” following which both groups were asked a class inclusion question (animals, with dogs as a major subclass). This was repeated with four concepts. In the first group more that one half of the participants committed at least one error but in the second group less that 10 % did. This is easily explained under the hypothesis that for the first group the preliminary question at best maintains the ambiguity and at worst invites to an exclusive interpretation (which will be transferred to the class inclusion question); whereas the second group are invited to answer “both”, suggesting an inclusive interpretation which they will transfer to the class inclusion question.

Other investigators have used procedures that help define the vocabulary used in the class inclusion question. This includes naming the classes (Inhelder et al. 1974), or explaining that the members of B’ are also members of A (Bideaud 1981). The disambiguation can be obtained even more explicitly by agreeing with the children to give a new name to the superclass (e.g., “round balls” for a set of blue marbles (B’) and red marbles (B), while asking them to compare the round balls with the red marbles (Sheppard 1973).

### The typicality of the minor subclass

Inhelder and Piaget (1959) noticed that performance varies with the concepts used (animals, flowers, fruit…), a phenomenon called “horizontal décalages” in their theory and which received *ad hoc* explanations in such terms as familiarity or abstraction.

Carson and Abrahamson (1976) manipulated the typicality of the subclasses. For example, they compared questions in which the minor subclass was atypical (e.g., five dogs and three bees: “more dogs or more animals?”) with questions in which the minor subclass was typical (e.g., five flies and three horses: “more flies or more animals?”). The performance was consistently higher in the first case than it was in the second. Similar results were reported by Lane and Hodkin (1985). The ambiguity hypothesis offers a straightforward explanation. In the first case, referring to *bees* by using “animals” is countermanded by the lack of typicality of *bees*, whereas in the second case referring to *horses* by “animals” is invited by the typicality of *horses*.

### Quantifying

To make the question sound more natural, Shipley (1979) presented 6- to 9-year-old children with a modified class inclusion question such as, “Which is more, only the lions or all the animals?” Children tested in a within-participant design improved their performance by one third. This result was confirmed by Hodkin (1981) who asked, “Are there more B or more of all the A?” in a between-participant design. She too attributed the improvement to the conformity of the sentence with natural language. Obviously, this explanation is circular, as the problem is to know why in everyday usage one would modify the superclass in this way. In fact, it is not infrequent that for the standard class inclusion question the older children spontaneously ask “do you mean all the A?” For a speaker who wishes to refer to the superclass and is aware that the hypernym can refer to the subclasses as well, the most economical way to communicate her intended meaning is to mark the union of the subclasses by “all”: The quantifier enables the speaker to refer unambiguously to the union of the subclasses and therefore to contrast the superclass with any one subclass.

### Qualifying

Wilkinson (1976) used materials modified as follows. All the members of A (houses) had a common perceptual feature (a window) and all members of B had another common feature (a door). This yielded three houses (A), two with a window and a door (B) and one with a window but no door (B’). The question was, “Are there more houses that have a door or more houses that have a window?” The performance of kindergarten children increased by 50 % when compared to a standard question with usual materials (children: two boys, one girl). Similar results were obtained by Dean et al. (1981) among 5- to 7-year-olds.

Similarly, McGarrigle et al. (1978) gave a qualifier to all the members of A, which introduced a second salient feature that should compete with the first (the one that defines the contrast between B and B’) and thus discourage exclusive comparisons. Six years old children performed better with such a material made of four lying cows, three black, one white (“Are there more black cows or more *sleeping* cows) than they did with the standard question (more black cows or more cows?”). This effect is explanable if one considers that there is a clue for disambiguating in favour of the superclass. Indeed, it should be noticed that the sleeping property does not appear in the definition of B (black cows). By contrast, the class to which the latter is compared in the question (sleeping cows) is described by the sleeping property so that its denotation as *all the cows* (because they all are sleeping) is encouraged. A control condition is missing, namely one in which the question would be “more *sleeping* black cows or more sleeping cows”, which presumably would produce a reduced effect, or even no effect. Generally, the existence of a feature perceptually salient common to all the A should enhance performance by helping to disambiguate in favour of the A when this feature qualifies only the hypernym in the question. The effect of saliency was demonstrated by Tatarski (1974) who presented 5- to 8-year-old children with three kinds of wooden blocks. The first set consisted of six cylinders wholly coloured (four blue, two red: “more wooden blocks or more blue blocks?”); the second set were painted over one half of the surface and the question was the same; the last set were wholly bi-coloured (four blue and yellow, two red and yellow: “more yellow blocks or more blue blocks?”). The rate of success increased significantly from the first set (below 50 %) to the second (below 60 %) to the third set (above 80 %), the increase from the second to the third reflecting nicely the increase in saliency of the common feature.

### The level of specificity

McGarrigle et al. (1978) report interesting results with non included classes. For instance, given cows (two black, two white) and horses (three black, one white) most 5-year-old children failed the question, “Are there more black horses or more cows?” Their spontaneous justifications suggest that they consider black horses and *black* cows. These observations were replicated and extended by Grieve and Garton (1981). They presented 4-year-old children with either equally or unequally specified questions. Instances of the former case are “Are there more black horses or more black cows?” for between-class comparison and “more black horses or more white horses?” for within-class comparison. These yielded near perfect performance. Instances of the latter case are “more black horses or more cows?” for between-class comparison and “more black horses or more horses?” (that is, the standard class inclusion question) for within-class comparison, which both yielded near complete failure. This was linked with exclusive comparisons, as could be inferred from children’s comments. For the between-class comparisons, the children introduced the qualifier when there was none. In brief, children treat the two sub-classes at the same level of specificity.

The results for the between-class comparisons were replicated with even greater accuracy by Gold (1984) who requested 5- to 9-year-old children to justify their responses to similar questions. Among those who failed questions such as “more black horses or more cows”, one third qualified *cow* by *black*, one third removed *black* from *horse*, 10 % added *white* to *cow*. Again, all these transformations amount to choosing comparisons at the same level of specificity. As McGarrigle et al. (1978) remarked, this strongly suggests that the source of the difficulty of the class inclusion question does not lie with inclusion, as the same kind of comparison is made for the between-superclass and the within-superclass cases. Children have expectations for comparisons that do not match the experimenter’s.

Based on these results, Shipley and Kuhn (1983) posited the equality in the level of specificity as an explanatory principle for class comparisons. They hypothesised that there exists a constraint on the selection of the criteria for membership in a class—which they call “target”—which accounts for the formation and consequent comparison of the wrong classes. The constraint, called “equally detailed alternatives” is that the set of targets corresponding to the classes being compared are specified in equal detail. This means, for instance, that if a value for colour appears in one target, some value for colour must appear in the other target(s). If a target is red square, the other target must specify a colour and a shape. If the experimenter’s description does not respect the constraint, the children form a different target by adding or eliminating some criteria, so that the classes that they compare are not those meant by the experimenter. Taking an example with natural kinds, in the request to compare poodles and dogs, the breed is specified in one target; by the constraint it must be specified for the other target, so that the child will compare poodles with a class homogeneous in breed.

The equally detailed alternatives hypothesis has the interest that it applies to comparisons of included and non included classes as well and it seems to be descriptively accurate. However, it is somewhat obscure as an explanatory principle, as it lacks a justification. It also has a limited explanatory power, as it cannot account for a number of effects already mentioned, such as the nouns of collections, counting, quantification, or typicality—indeed the authors acknowledge that the constraint is not the only source of difficulty. Moreover, it seems to lack parcimony as from the present viewpoint this hypothesis is derivable from considerations of relevance. As the authors noted themselves, “specifying ‘red’ for one class has made color relevant to membership in all classes. Essentially, this is the equally detailed alternatives hypothesis” (p. 200). In this quote, they used the expression “relevance” in a pre-theoretical sense. Theoretically, specifying the value of a feature establishes a presumption of relevance of this feature to refer to the classes mentioned in the dialogue or at least in the utterance. If the speaker takes the trouble to specify the value of an attribute for one class, this creates the expectation of being informed about the value of this attribute for the other class; and if this value is not pertinent to refer to the other class, this is normally marked by “all”. For instance, given short and tall green trees and short and tall brown trees, “the short green trees or the brown trees” is less felicitous than “the short green trees or *all* the brown trees”. In fact, in agreement with this analysis, the five-year-old children tested by the authors did commit more errors in comparing the short green trees and the brown trees given these four classes than they did when they were given short and tall green trees and wide and narrow brown trees. We conclude that the equally detailed alternatives hypothesis can be considered as an accurate description of phenomena that are explanable within the pragmatic framework.

### Mentioning both hyponyms in the question

Winer (1978) asked pairs of questions of 8- to 10-year-old children. These combined a request for an exclusive comparison (“more dogs or more cats?”) and a standard question (“more dogs or more animals?”), which resulted in a higher performance than that of a control group. A likely explanation is that the possibility for the hypernym to refer to the minor subclass of cats is blocked by the use of the minority hyponym (cats) to refer to it. Ahr and Youniss (1970) used all three nouns in the question (“more animals, or more dogs, or more cats”) and observed a significant improvement, explainable by the same mechanism. Unhappily, their participants had already received a class inclusion question, and the novel questions were formulated with “less” or with “more”. But in the latter case the exclusive and inclusive comparisons are indistinguishable because both lead to a correct answer, so that the source of the overall improvement is not clearly identifiable.

There is, however, evidence that the mention of all three nouns enhances performance. This comes indirectly from the investigation of the so-called “verbal facilitation” described by Wohlwill (1968). He observed higher performance when the class inclusion question was presented only verbally without pictures or objects, which was replicated by Winer and Kronberg (1974) at all ages from 6 to 11, and by Padilla and Romero (1976) with 9- and 11-year-olds (but Cameron and Goard (1982) failed to replicate this effect). As noted by Winer (1974) the strict verbal presentation is accompanied with additional verbal cues, namely the mention of the minority hyponym. In fact the question posed was always of the type “if I had four apples and three pears, would I have more apples or more things to eat?” That the facilitation stems from this confounding factor rather than from the absence of material is supported by the absence of difference in performance between a group that received the modified question without material and another one with material. Another confirmation comes from a study by Brainerd and Kaszor (1974) who failed to replicate the effect when using a formulation in which the minority hyponym did not appear (“Are there more red circles than there are circles?”) and the picture was turned face down.

In brief, it seems that the mention of the three class nouns does improve performance and the reason is clear. The hypernym is less likely to refer to B’ (and consequently more likely to refer to A) when the minority hyponym which refers to B’ is used in the sentence: In other words, this helps disambiguate the sentence.

The role of the minority hyponym was subsequently discussed by Agnoli (1991) within the conceptual framework of representativeness. She presented 9-, 11-, and 13-year-old children with class inclusion questions without material. There were two question types that differed by the representativeness of the major subclass, such as: “In summer on the beach, are there more ladies or more tanned ladies?” versus “…or more pale ladies?” The rate of errors was 62 % and 28 %, respectively, which coincides with choosing the representative class *(tanned ladies)* in the first case and avoiding the non representative class *(pale ladies)* in the second case. However, these results, which reproduce and generalise those obtained by Carson and Abrahamson (1976) and Lane and Hodkin (1985), are explanable linguistically as the author noted. If *ladies* tends to refer to the complementary subclass B’, an incorrect response is more probable when the hyponym mentioned is *tanned ladies* (the complementary subclass *pale ladies* is less numerous) than when it is *pale ladies* (the complementary subclass *tanned ladies* is more numerous). The author tried to test this hypothesis by adding a question with all three nouns. The results indicate a persistent preference for the representative answer but the within-participant design of the experiment makes the result hard to interpret. The effectiveness of this kind of modified question will be demonstrated in “The factors that affect performance” section.

### Learning inclusion

A variety of learning procedures have been shown to improve performance. Simple repetition with feed-back is one of these (Ahr and Youniss 1970; Brainerd 1974; Siegel et al. 1978; Youniss 1971). This is not surprising as following negative feed-back the child will tend to change interpretation by changing the reference of the hyponym.

Judd and Mervis (1979) asked 5-year-olds to count the objects in the superclass and the subclasses (three toys, two balls, one bear), after which the class inclusion question was posed and the counting repeated if the answer was incorrect, and again until success. After this training a new class inclusion question was asked as a posttest where the rate of success exceeded 80 % against just a few percent in a pretest. This increase was attributed by the authors to the contradiction between the result of the correct counting (three toys, two balls) and the incorrect answer (more balls than toys) which finally the children must become aware of. However, no precise description of the process that leads to the answer is proposed. A likely explanation is that the child is offered an occasion to disambiguate the reference of “toy”: The hypernym initially refers to the bear in the question, but to the superclass when counting so that in the end it is given the intended reference. In other words, the counting and training procedure enable the child to learn the experimenter’s use of the names.

Kohnstamm (1963) was even more directive in explaining that “there are more A because B are also A. B and B’ are all A and so there are always more A”, or “they are all A and only two are B”, etc. following which most children aged 5–7 were successful.

In sum, a learning method for the inclusion task is effective if it enables the child to realise that the intended comparison is that of the major subclass to the superclass. All these methods have in common that in the end the child has learned the experimenter’s use of the words, that is, the hypernym refers to the superclass and not to the minor subclass.

## Testing the pragmatic approach

In the previous section we have examined the hypothesis that the referential ambiguity of the hypernym is what makes possible children’s exclusive comparisons and we have shown that it has strong explanatory power. But to establish the explanation of the performance that we propose, we need empirical evidence supporting two of its claims.

### Young children’s referential attribution of the hypernym

The first claim, which is implicit, is that the younger children do understand the referential properties of class names, that is, know that the hypernym can also be used to refer to a subclass. This was demonstrated by Smith and Rizzo (1982). In a first experiment, 4- and 5-year-olds were presented with materials such as three daisies and three roses and requested to tell whether a puppet named objects correctly or not (e.g., flowers for the roses, flowers for all the flowers, roses for the roses). About two thirds of the children accepted the reference of the hypernym to both the superclass and the subclass indicating knowledge of the referential properties of the hypernym.

The results of two other experiments support the notion that the hypernym is ambiguous. In a second experiment 5-year-olds were requested to get a set of objects, put it back and then get another set; this was done in the case of two subclasses (e.g., daisies then roses) and in the case of a superclass and a subclass (e.g., flowers then roses) by instructing the child to “get the—and then get the—”. Performance was virtually perfect in the first case but did not exceed 14 % in the second case, suggesting difficulty in attributing reference to the complementary subclass—however children may also fail because, as the authors acknowledge, the question requiring to take back some objects already taken is particularly tricky. In a third experiment one group of 5-year-olds was given the same task as in the second experiment while another group received this task with feedback. In addition, both groups received a class inclusion question as a pretest and as a posttest. The no-feedback group committed three times as many errors as the other, suggesting that the source of the errors is a lack of clarity in the reference of the hypernym, which was remedied by the feedback as the intended reference got progressively fixed across trials. Also the no-feedback group did not improve from the pretest to the posttest whereas the other group jumped from 20 to 75 % correct. This suggests that the training was effective in disambiguatng the hypernym. This work is important in showing that 5-year-old children know that a hypernym can refer to the subclass and to the superclass, and also in indicating—although indirectly—that the hypernym is ambiguous and that this can be overcome by a training procedure which helps disambiguate the hypernym.

### The subclass-to-subclass comparison

The other claim of the present approach, which is explicit, is that the younger children who fail the question make subclass to subclass comparisons. Starting from Piaget himself, there is unanimity in favour of this claim, with the only exception of Brainerd and Kaszor (1974). They based their denial on the results of one of their experiments in which they asked children to recall the question. They hypothesised that if children referred to the subclass by the hypernym, one should observe substitutions during recall (the child reformulating the question as “more B or more B’ ”) and such errors should be more frequent after an incorrect response. Because they found few cases of substitution and no differences in frequency in a condition with immediate recall, they rejected the hypothesis. This clearly is too hasty, for the hypothesis is based on the assumption that children should reformulate the question in the same terms that coincide with their interpretation. This is very doubtful as it is the experimenter’s role to define the task, give the instructions and fix the use of the vocabulary. If a child hears the name A and interprets it as referring to B’, he is likely to continue to use the experimenter’s word A to refer to B’, especially for an immediate recall.

This is borne out by results obtained by McCabe et al. (1982) who asked five class inclusion questions with various concepts and only then asked a recall of the questions: Among the 5-year-olds who answered incorrectly, the majority recalled the question in terms of the hyponyms. Further evidence of exclusive comparisons can be found in a study by Ahr and Youniss (1970) who varied the ratios of the number of items in the subclasses (dogs and cats). With eight dogs and no cat most 6- to 8-year-olds answered “more dogs” suggesting an unsuccessful search for cats. This interpretation is born out by the answer to the question formulated by “fewer”, which was “fewer animals” most of the time. Even more significantly, with four dogs and four cats the tendency was to answer “same” (half of the children to the “more” question and the great majority to the “fewer” question). Trabasso et al. (1978) offer further evidence in an investigation in which the standard question (“more A or more B”) was compared with a question of the type “more A or more B’ ”). Whereas the rate of success ranged from one third to two thirds, depending on age, it was always above 90 % with the second question. This is easily explained if the children make exclusive comparisons. B is always chosen because there are more B than B’; so, with the standard question B is denoted by the hyponym B and the children answer “B” whereas with the other question B is denoted by the hypernym A so that they answer “A”, which surreptitiously increases the rate of apparently correct responses. Naturally, the use of B in the formulation of the standard question is motivated to avoid this possibility. Interestingly, McCabe (1987) has shown that even adults may commit errors under time constraint. When requested to identify the question asked, subclass comparisons were falsely recognised 30 % of the time.

In brief, there is overwhelming evidence in support of the claim that participants actually perform an exclusive comparison between subclasses following the class inclusion question.

### Demonstration of the referential ambiguity in the standard question: experiment 1

The claim that the hypernym can be used to refer to the subclass as well as to the superclass will now be substantiated by demonstrating that the spontaneous reference attributed by children to a hypernym depends on whether or not it follows the mention of one of its hyponyms. No class inclusion question was asked in this experiment; there were only requests for designation.

#### Participants and material

Thirty children, aged 6;7 to 7;7 (median: 7;1) from a primary school in a small French city were presented with two kinds of concepts: Flowers (five asters and three tulips), and fruit (four bananas and three apples). For this and the following experiments the classes were drawn in colour on a Bristol board and the children were tested individually in an isolated room. Parents’ consent to the children’s participation was obtained through the school administration.

#### Design and predictions

There were two experimental conditions with 15 children in each. In the AB-BA condition the children were asked to designate the superclass (“show me the flowers”) by pointing with their finger; immediately after answering the children were asked to designate the subclass B (“show me the asters”). Then the same request was made in the reverse order with the fruit (“show me the bananas”, then “show me the fruit”). In the BA-AB condition the order of the requests was: Asters, flowers, then fruit, bananas. This design allows to vary the position of the crucial pair of requests AB (first vs second position) and the concepts (flowers vs fruit). Care was taken to let the children answer at their own pace and make exhaustive choices.

It was predicted that in response to an *initial* request for A (mention of the hypernym), the designated items would belong to both subclasses because a preference for any one subclass is irrelevant: Children will make an *inclusive use* of the hypernym. In contrast, when the same request follows a previous request to show B, then there should be cases where children designate B’ exclusively. This is because in the context of a previous request to show one subclass (B), designating the complementary subclass (B’) is now relevant as this materialises the partition and establishes B’ on par with B, which is at the same hierarchical level: If you have asked me to show one subclass, then it is reasonable for me to expect that the next request will be to show the other subclass. These are cases of an *exclusive use* of the hypernym.

#### Results and discussion

We are interested in the answers to the request to show the class A, and comparing this answer as a function of its position, before or after a request to show the subclass B. The results appear in Table 1 and they are clean-cut. Because there was no difference as a function of the type of concept, we consider the totals.

**Table 1:** Experiment 1

	The request to show the A was made			
	Initially		After a request to show the B	
	Flowers	Fruit	Flowers	Fruit
Child shows A (= B + B’)	14	13	9	7
Child shows only B	1	2	6	8

Number of choices upon request to show the members of the class for the two materials (flowers and fruit)

*A* superclass, *B* major subclass

Initially children were overwhelmingly correct in showing the A (B + B’), but in the context of a previous request to show the B now about one half showed only the B’ (and the other half the B and the B’). The differences in the numbers of choice are significant for both concepts (Fisher test, p < .05). In brief, the reference of name B has become fully ambiguous between the complementary subclass B’ and the whole class A. Interestingly, following the choice of B’, a few children interrupted themselves (with their hand hovering above the drawing) and then carried on to complete their choice with B, an hesitation which nicely reveals the ambiguity.

The consequence for the formulation of the class inclusion question is straightforward: Because the names A and B are mentioned in the same sentence, the tendency to interpret A as referring to the B’ should be even stronger than it was in the experiment where the names A and B occurred in two separate sentences. Based on the notion that the standard class inclusion question is ambiguous, and having identified the origin of the ambiguity, the next step now is to construct a *modified* class inclusion question devoid of ambiguity to get the correct performance on the simple judgement of inclusion.

### Elaborating a modified question: experiments 2 and 3

A modification to the standard class inclusion question suggests itself, namely mentioning the superclass and the two subclasses in the question. As reported ealier, this was already done by Ahr and Youniss (1970) and by Agnoli (1991), but with inconclusive results. Experiment 2 was designed to test the effect of this manipulation.

#### Participants and materials

For this and the next experiments, the participants came from a suburban residential area near Paris. Forty-two kindergarden children aged 5;1 to 6;0 (median: 5;6) from a kindergarden were presented individually with two kinds of concept: Fruit (five pears and three bananas) and flowers (four tulips and two asters).

#### Design and predictions for experiment 2

Each child was asked only two questions, one standard (henceforth the standard question), the other modified (the modified question). There were two conditions, with 21 children in each, that served as mutual control and differed by the order of the questions: standard question first or modified question first. The use of the two concepts (fruit and flowers) was counterbalanced. This design allows both within- and between-participant comparisons. Before both questions the experimenter made sure that the children knew the reference of the subclasses by requesting an initial designation; there was an additional request to designate the superclass before the modified question. The questions were, “Are there more B or more A?” for the Standard Question, and “Are there more B or more B’ or more A?” for the modified question. No feed-back was given after the child’s answer.

It was predicted that performance between- and within-participants would be higher on the modified question than on the standard question because the former question is disambiguated as the references of A, B and B’ have been fixed by designation and by the mention of all three names in the question, so that the hypernym must refer to A and the major hyponym to B.

#### Results and discussion

Table 2 presents the cross-distribution of the answers.

**Table 2:** Experiment 2

	Order		Standard question		
			Failure	Success	Total
Modified question	Standard–modified	Success	6	1	7
		Failure	12	2	14
		Total	18	3	21
	Modified–standard	Success	2	8	10
		Failure	11	0	11
		Total	13	8	21

Cross-distribution of the answers to the two questions for the two orders

The between-participant analysis performed on the question presented first shows that three children (14.3 %) passed the standard question (a usual rate for the present age range) compared to 10 (47.6 %) who passed the modified question, an unusually high rate; this difference is significant (Chi square = 5.70, p < .01). The higher performance is confirmed by a nearly significant result within participants: Eight children passed the modified question and failed the standard question against two who had the reverse pattern (binomial test, p = .055). Finally, considering success on the standard question, it appears that 3 children (14.3 %) passed it when presented before the modified question against 8 (38.1 %) when presented after; this is a significant difference (Chi square = 3.07, p < .05) indicating that the Modified Question helps improve performance on the standard question: In receiving the first question some children learned that the hypernym does not refer to the subclass and transferred this to the standard question.

Children’s reaction time to the request to designate the superclass after their designation of the two subclasses was most suggestive. Whereas the reaction to designate the subclasses was generally immediate, the time to designate the superclass (which came after designation of the subclasses) was typically several seconds; in fact, the experimenter often needed to amend the question (“show me *all* the A”) for the child to answer.^3^

In this experiment the modified question was highly effective in increasing performance. Now because a request for designation accompanied the mention of the hypernym, one may question whether the sheer mention of the hypernym is sufficient to improve performance. The next experiment was designed to answer this question.

### Experiment 3

#### Participants, design and materials

The materials, design and procedure were the same as for experiment 2. The participants were fifty-one children aged 5;10 to 6;11 (median 6;5) coming from a primary school in the same residential area. The two questions were again a standard and a modified question. However this time both were preceded by requests for designation. In brief, the two tasks differed only by the presence or the absence of the minority hyponym (B’) in the question. It was predicted that performance would be higher with the modified question than with the standard question because the formulation of the modified question disambiguates the hypernym.

#### Results and discussion

Table 3 presents the cross-distribution of the answers. The between-participant analysis performed on the first of the two questions shows that, as expected, performance was higher with the modified question than with the standard question, as the number of correct answers were 20 (80 %) and 14 (53.8 %) respectively, which is sigificant (Chi square = 3.91, p < .05). This result is confirmed by the second of the two tasks (88.5 and 48 %, respectively). It is also confirmed by the within-participant analysis which indicates a highly significant effect of the modified question: 18 children passed it and failed the standard question against only one who passed the standard question but failed the modified question (McNemar test, Chi square = 14.22, p < 5.10^−4^). These results still obtain for each order of presentation separately (McNemar test, Chi square, p < .01).

**Table 3:** Experiment 3

	Order		Standard question		
			Failure	Success	Total
Modified question	Standard–modified	Success	10	13	23
		Failure	2	1	3
		Total	12	14	26
	Modified–standard	Success	8	12	20
		Failure	5	0	5
		Total	13	12	25

Cross-distribution of the answers to the two questions for the two orders

In sum, there is a definite advantage in adding the minority-hyponym (B’) in the question, as predicted. It is not clear why this manipulation failed in Agnoli’s (1991) experiments.

The discrepancy may stem from a difference in the order of the three terms in the question. In experiments 2 and 3 the hypernym always came last, whereas its position was counterbalanced in Agnoli’s main experiment (and there is no information for the additional experiment). Another difference is that in experiments 2 and 3 the question was preceded by a request for designation. It is now important to separate the respective importance of the request for designation from the presence of the hyponym in the question in the disambiguation. In addition, we wish to get the developmental trend. The next experiment will attempt to fulfill these objectives by presenting children aged 5–8 with four tasks: The Standard Question and the Modified Question, both with and without a previous request for designation.

### The developmental trend: experiment 4

The results of experiment 2 suggest that children as young as 5 or 6 years old could pass the question if it was properly interpreted. Consequently in experiment 4 the age range started as early as 4;6 (finishing at 8;9).

#### Participants and materials

The participants were 386 children from kindergarden and primary schools. The age ranges were 4;6 to 5;5 (N = 59); 5;6 to 6;5 (N = 138); 6;6 to 7;5 (N = 123) and 7;6 to 8;9 (N = 66) with median ages of exactly 5;0, 6;0, 7;0 and 8;0, respectively. Two concepts were used: Fruit (five pears, three bananas) and animals (four lions, two elephants).

#### Design

The children were presented with two tasks in four conditions as follows:

Condition I: (1) Standard question. (2) Modified question after request for designation of the three classes.

Condition II: (1) Standard question after request for designation of the three classes. (2) Standard question.

Condition III: (1) Modified question. (2) Standard question.

Condition IV. (1) Modified question after request for designation of the three classes. (2) Standard question.

Condition I was an exact replication of one of the conditions of experiment 2. Condition IV differed by the exchange of the order of the two tasks. The first task in condition IV cumulates the disambiguations introduced in the first task of conditions II (designation) and III (modification). In all the conditions the two concepts were used in counterbalanced order.

Conditions II and III were administered to the 5- and 6-year-olds only. Because I was a control and IV the target condition these two were administered to all four age groups.

#### Predictions

We begin with the first task. Performance should be higher in condition IV (which cumulates two disambiguating procedures) than in conditions that have only one (III and II) or none (I); the latter two comparisons predict replications of the effects observed in experiments 2 and 3. Also performance should be higher with either of the two ways of disambiguating the standard question: By modification of the question (we expect III > I) or by a request for designation (we expect II > I). In brief, the predictions for the performance on the first task can be summarised by five inequalities : IV > I; IV > II; IV > III; III > I; II > I. Notice that no prediction is made between conditions II and III: It is an empirical question to know which of the two disambiguating procedures is the most efficacious.

The second task aims to test a secondary hypothesis: A transfer effect as observed in experiment 2 would result in higher performance on the second task in conditions II, III and IV.

#### Results and discussion

Table 4 presents the percentage of correct responses. All the comparisons that follow are statistically significant using Chi square tests at p < .05 (most of them well beyond this level). We begin with the first task.

**Table 4:** Experiment 4

	Task			
	Request for designation			
	Standard question		Modified question	
	Absent	Present	Absent	Present
Condition	I	II	III	IV
Age				
5	6.6			52.0
6	5.9	28.5	25.7	55.9
7	18.7	61.3	38.7	96.6
8	42.4			97.0

Response frequencies in percent

The results of experiment 1 are confirmed and generalised: The comparison of columns I and IV shows that by combining the two disambiguiting procedures there is a spectacular improvement in performance across all ages. In particular for the 7-year-olds, the rate of success jumps from less that 20 % to near perfection. Also for the 5- and 6-year-olds, the conventional criterion of inclusion (more than 50 % success) is reached. Recall that this is usually attained between 8 and 9 years. Importantly, the rates in condition I are typical of the common results, so that the possibility that the children were particularly advanced in their development can be ruled out.

Next, comparison of column I with columns II and III shows that each disambiguation procedure was effective separately. It was effective to roughly the same extent for the 6-year-olds but for the 7-year-olds the request for designation was the most effective. Finally, comparison of column IV with columns II and III shows that performance is higher when both procedures of disambiguation are cumulated rather than using any one alone.

We now consider the second task. We first relate performance on the standard question when it is asked first and when it is asked second; this is a between-participant comparison. The percentages of success are 28, 29.8, 62, and 84.8 % for the four age groups respectively, to be compared with the figures in the first column of Table 4: 6.6, 5.9, 18.7, and 42.4 %. This indicates a very important transfer effect, showing that children have learned the rule of the game, so to speak, on the first task, that is, the conventions used for the names to refer to classes and then apply this subsequently in the second task.

The within-participant analysis is based on Table 5 which presents the cross-distribution of answers when the second task is a standard question. Averaging across the ten 2 × 2 sub-cells, it appears that (i) failure at the disambiguated question almost always implies failure at the standard question (in 94 % of the cases) and this applies at all ages; (ii) success at the disambiguated question most generally implies success at the standard question (in 81 % of the cases) with he exception of the younger children. It is again apparent that cumulating both disambiguating procedures is conducive to the best transfer, followed by the request for designation, which in turn is more efficacious than the modified question.

**Table 5:** Experiment 4

	First task: question type						
	Standard prepared by a request for designation			Modified		Modified and prepared by a request for designation	
Condition	II			III		IV	
	+		−	+	−	+	−
Second task (standard question)							
Age							
5	+					8	0
	−					7	14
6	+	8	1	6	1	13	1
	−	2	24	3	25	6	14
7	+	18	1	10	2	26	0
	−	1	11	2	17	2	1
8	+					28	0
	−					1	4

Cross-distribution of answers to the two tasks when the second task is a standard question

+ Correct, − Incorrect

Because the hypernym is ambiguous, as long as it is optimally relevant for the children to opt for an exclusive interpretation, they will compare the two subclasses. The results of experiment 4 have established that when care is taken to formulate the simple class inclusion question in a way that disambiguates in the intended sense, children as young as 5 years old can pass it because now they can engage in the comparison intended by the experimenter. The results show that *the simple judgement of inclusion is made correctly three to four years earlier than is usually claimed in the literature*.

There is, however, one possible methodological objection to the results of experiments 2, 3 and 4 that concerns the modified question. Because the modified question has been formulated with the hypernym in the last position, couldn’t it be the case that the improvement in the performance reflects only an order effect? This means that the child would choose response A more often just because A appears the last in the question. There is some pertinence in such considerations as an order effect was observed with the standard question (Kalil et al. 1974): The order B, A yielded higher performance than the order A, B. However, the hypothesis that order is the only factor of facilitation must be rejected because in our experiments the standard question too has been formulated in the B, A order. So, if the child followed a heuristic to select the class whose name is the last, performance should be the same with both questions, but this is not so; consequently there is more in the effectiveness of the modified question than just an effect of order that would reflect a heuristic based, e.g., on an expectation that the experimenter keeps the correct option at the end of the sentence. However, the existence of an order effect with the standard question is intriguing in itself. These considerations lead us to a refinement of the linguistic analysis that we now develop.

### More on the psycholinguistic analysis of the question: experiment 5

In the formulation of the modified question the hypernym A was placed at the end on purpose. Indeed, the order of the names is not indifferent from the viewpoint of the linguistic theory. When both hyponyms B and B’ have already appeared in the sentence, the hypernym A is unlikely to be given the same reference as B or B’ because the extension of the subclasses has already been denoted; this optimises the exploitation of the use of B’ to disambiguate A. But if A appears before both B and B’, there are a number of possibilities such as deferring reference until after B and B’ have been mentioned, or give A a revocable reference that may or may not be revoked at the end: The final assignment of A to B’ is not so straightforward and less warranted. Experiment 5 was designed to test the hypothesis that performance is affected by the position of A in the question.

#### Participants and materials

Seventy-one primary school children aged 5;10 to 7;0 (median 6;4) were presented with the fruit drawing (five pears and three bananas).

#### Procedure, design, and predictions

One single question was asked, preceded by a request for designation. The order of the three names in the question was varied according to all six possible permutations constituting six groups of 11 or 12 children:

(1): B’ B A (2): B B’ A (3): B’ A B (4): B A B’ (5): A B’ B (6): A B B’

We have seen that the best performance is expected to occur when A is the last mentioned. When it is not, there is an additional treatment and a load in working memory which is costly, especially for the younger children. As a first approximation, we hypothesise that the difficulty is an increasing function of the distance of A from the end position. Thus, the prediction for the correct response rate is: B’ B A = B B’ A > B’ A B = B A B’ > A B’ B = A B B’

#### Results and discussion

Table 6 presents the numbers of answers for each group. When the position of A is kept constant within the three sub-groups (last, middle, first) the frequency of A answers does not vary. The comparison between the three groups obtained by collapsing (1) and (2), (3) and (4), and (5) and (6) indicates that the position of A is the only factor that yields a variation in the frequency of A responses, with the lowest rate for the first position but the middle and first position yield equal rates, contrary to the prediction of a decrease from last to middle. However, the whole trend is compatible with the prediction of a *general* decrease (Jonckheere trend test for ordered alternatives, z = 2.06, p < .05).

**Table 6:** Experiment 5

Order and group number	Position of A					
	Last		Middle		First	
	B’BA 1	BB’A 2	B’AB 3	BAB’ 4	AB’B 5	ABB’ 6
Answer						
A	7	6	6	7	2	3
B	4	5	6	5	9	9
B’	1	1	0	0	0	0
Total	12	12	12	12	11	12

Frequency of answers as a function of the position of the hypernym A in the question

So, putting A in the mid-position resulted in as much improvement as putting it last. This is compatible with the post hoc hypothesis that the contiguity between A and the last hyponym is necessary for A to remain in working memory and have better chance of receiving its correct reference, whereas in the first position A is readily lost. Of course, this interpretation needs independent experimental support.

### Conclusion of experiments 1–4

The experiments reported offer direct evidence that in the standard class inclusion question the hypernym (A) has referential ambiguity (of the privative variety). Experiment 1 has shown that it can refer with an inclusive denotation to the superclass, but also with an exclusive denotation to the subclass that is not mentioned in the question, that is, the minor subclass B’. The main claim of this paper is that the interpretation of the hypernym is pragmatically determined as a function of the child’s perception of the aim of the standard task, which evolves with age. Depending on their level of development, children may or may not adopt spontaneously the interpretation that enables the experimenter to test their acquisition of the simple inclusion judgement. One interpretation (the exclusive one) does not offer this possibility. Consequently, experimenters who wish to know whether the younger children are capable of the simple inclusion judgement should attempt to disambiguate the hypernym and help interpret the question in such a way that the hypernym refers to the superclass, which is its intended meaning in the standard question; only then can it be considered that the children are put to a valid test. The results of experiments 2, 3, and 4 have shown that when one, or even better, two disambiguation procedures are applied, the children reach the critical behavioural criterion of inclusion three to four years earlier than is usually claimed in the literature, that is, as early as five years of age. This is by no means a lower bound, rather it may be the limit that the present means of investigation is able to reach.^4^

## Two other approaches to the task

In this section we consider two theoretically motivated explanations of performance on the class inclusion task and show their inadequacy.

### Experimental investigation of the role of collections

We have mentioned earlier (“Classes versus collections” section) that facilitation was observed when the name of a superclass is replaced by the name of a collection and we have offered a linguistic account of it. Here we take a closer look at this phenomenon and we test the linguistic explanation against an explanation based on the internal organisation of collections and their psychological coherence.

#### Degrees of internal organisation

In a number of studies devoted to the comparison of collections and classes (Markman 1984; Markman and Seibert 1976) has emphasised the existence of the following differences. The first one concerns the part-whole relationship: It is a *part of* relationship in the former case but an *is a* relationship in the latter case. Further, to determine membership in collections one needs to know something about the relationship to other members, which is unnecessary for classes. Finally, in the same way that the various parts of an object are organised to constitute the whole, a collection has at least some degree of organisation, e.g., spatial or temporal like in a family, a crowd or a pile. All this contributes to the child’s better conceptualisation of holistic properties for collections than for classes, which in turn should make part-whole comparisons with collections easier than with classes.

Even though the experiments of Smith and Rizzo (1982) have demonstrated that children know that the hypernym can also be used to refer to a subclass, these authors did not exclude Markman’s hypothesis that collections have internal organisation that can facilitate the inclusion task. Further, they questioned the feasibility of empirically separating the contribution of organisational properties of collections from their referential properties. We take up this challenge now.

Markman and Seibert (1976) considered the internal organisation as a matter of degree. While they used the degree of organisation as a variable opposing objects and collections, this may be used as a within-collection variable: Indeed it is clearly in the spirit of their theory to assume that different degrees of organisation should result in different degrees of psychological coherence. For instance, a tribe seems to have greater organisation than a crowd. Is it possible to define criteria to assess such degrees of organisation?

To answer this question, we propose four criteria. The first two are linked to the nature of the relationships that constitute the collection. These relationships are necessarily verified by any member of the collection, and must be distinguished from the membership relation *(is a part of)*. They can be characterised by their strength and their number.

1. The strength of a relationship is a function of (i) the temporal stability (persistence over time): It is higher for a village than for a bunch of flowers; (ii) the spatial stability. It is all the greater as the relationship is independent of space, that is, resists to dispersion: It is higher for a family than for a bag of marbles; and (iii) the number or proportion of members that verify one or more relationships.
2. The number of types of relationship. It is higher in an orchestra than it is in a packet of cigarettes. The two other criteria characterise the members of the collection.
3. The permanence of the members. The higher their duration, the higher the coherence of the collection. It is higher for a forest than for a basket of fruit.
4. The existence of a function specific to the collection. Generally, each member does not individually possess this function but contributes to it. It is what justifies the collection. A deck of cards must be complete for a given game to be played according to the rules; at the other end, some collections only cumulate members to reach an amount, as in a pile of plates. Of course, some members may have an individual function, but this is linked to the number of relationships: For instance, the tea pot has an individual function in the tea set and at the same time defines a specific relationship with the other members (“pouring into”).

In brief, it is possible to separate the referential properties of collections (the membership which is an all-or-nothing property that they all possess) from their organisational properties, which are variable and susceptible of an objective determination. Markman’s theory is thus testable: It will suffice to define collections of various coherence, and compare performance on the class inclusion question using these different collections. The theory predicts that performance will increase with the degree of coherence. By contrast, the linguistic approach predicts no difference because the only pertinent factor is the referential properties, which are invariant across collections.

#### Experiment 6

##### Materials

To design weak and strong collections, a list of 24 collections was submitted to 10 academic staff members in a psychology department who served as judges. They received instructions that detailed the four criteria defined above and were asked to rate each collection on a five-point scale of strength. For the sake of simplicity, it was decided to keep only two levels of coherence, defined by the four weaker and the four stronger collections on the scale. To do so, two statistical criteria were chosen. One, a central tendency criterion: The mean rating must be <2 for a collection to be considered weak and >4 to be considered strong. Two, a variability criterion: No collection was accepted as weak if any one judge gave it a rating above 3; and no collection was accepted as strong if any one judge gave it a rating below 3, which is very demanding as it means that it required strict unanimity. This resulted in the following eight collections: Pack of candies, bag of marbles, row of cubes, pile of plates (weak); tribe of Indians, team of volleyball, jazz orchestra, family of cats (strong). Pictures of these were drawn, taking care that features such as number of members and disposition be equally balanced across weak and strong collections because these factors had to be controled for the class inclusion question.

##### Participants, design, and procedure

Thirty-six children aged 5;0 to 5;11 (median 5;6) from a kindergarden were presented with the eight pictures (4 weak, four strong collections); they acted as their own controls. The order of presentation was counterbalanced with regard to rank in the whole series and contiguity of weak and strong collections. For each picture the children were requested to point to the major subclass, the minor subclass and the collection, following which they were asked the class inclusion question, e.g., “You will have more cats if you are given the kittens or if you are given the family?”

##### Results

The rate of errors was virtually equal for the weak (27.1 %) and the strong collections (28.5 %). The distribution of the number of errors was the following:

Weak collections. Total = 39 (candies = 8; marbles = 9; cubes = 8; pile = 14).

Strong collections. Total = 41 (tribe = 7 team = 11 orchestra = 13; family = 10).

Individually there were no differences in performance either: Twelve children committed more errors with weak collections than they did with strong ones; thirteen committed fewer errors; and 11 committed as many errors.

In brief, contrary to a hypothesis derived from Markman’s theory, collections that were sharply contrasted from the viewpoint of their degree of psychological coherence were treated identically by children in the class inclusion task. But this is in agreement with the linguistic claim: The class noun allows reference to the minor subclass, but the collection noun does not, which eliminates the referential ambiguity.^5^

In fact, other experimental results cast doubt on the psychological reality of the coherence concept. If such a notion did affect children’s conceptualisation of sets, then performance would be enhanced also on other tasks, such as number conservation and cardinality; but this claim initially made by Markman (1979) was not confirmed by later studies (Fuson et al. 1988; Hodges and French 1988).

### The fuzzy trace theory

The fuzzy trace theory of class-inclusion (Brainerd and Reyna 1990; Reyna 1991) may be the most sophisticated approach to this task. It has the advantage of being part of a wider theoretical background that applies to various reasoning and judgment tasks.^6^ The main claim of the theory is that mature inclusion reasoning is not quantitative but qualitative; it is pattern based and therefore nonnumerical. The patterns are the result of the extraction and storage of a type of gist, namely the familiar relationship of inclusion hierarchy; then they are processed by the application of a qualitative ordering rule, namely the cardinality principle which states that the more inclusive of two sets is necessarily the more numerous. For a mature individual, there is an algorithmic procedure to answer the class inclusion question: Encode and store the gist (“there is an inclusion relation”), retrieve the cardinality principle in memory, and apply it, which is little demanding. When solving the task, the child encodes both the inclusion gist (e.g., cows are animals, horses are animals) but also the “relational” gist (that is, the exclusion relation: There are more cows than horses). Encoding or storage are not a source of difficulty. The source of erroneous responding is that the relational gist is more salient, hence a tendency to judge relative numerosity instead of applying the cardinality principle. In the inclusion task there is a difficulty specific to the comparison of the numbers of A and B because this requires keeping the whole in memory while one of its parts has been separated. (So the theory applies to numerosity at a processing level the analysis that Piaget applied to sets at a logical level). The inclusion relation, even though it is understood, is implicit whereas the “relational” relation is visible and explicit. The two relations compete and failure occurs when the latter dominates the former. This immature reasoning may take place even if the child possesses the cardinality principle. In brief, failure reflects a defective performance, not a lack of competence. For the younger children there may be a retrieval failure, and for the older ones a processing failure. In this latter case, there is difficulty fitting the cardinality principle to memory for the inclusion gist. This is why it is expected that cuing members of the superclass with a distinctive tag makes the hierarchical levels more separable. The processing of the principle will be more accurate and a higher performance is expected.

Brainerd and Reyna (1991) tested the latter prediction in several experiments (inspired by Wilkinson 1976, and by Dean et al. 1981) in which the salience of the members of the superclass was manipulated. These will not be described here for lack of space. We note that all the results can be accounted for within the pragmatic framework. For instance, the manipulations that increase the salience of the members of A (e.g., red) together with a qualification of the hypernym (the red A) are equivalent to those discussed earlier (see section on qualifying): They are genuine procedures of disambiguation which increase the likelihood that the hypernym refers to all the A.

One may wonder whether, reciprocally, the effect of all the sucessful manipulations described in “The factors that affect performance” section and the novel ones described in “Testing the pragmatic approach” section can be explained by the fuzzy trace theory. The answer seems negative. Take for instance the effect of the typicality of the minor subclass on performance. Introducing an atypical subclass keeps the irrelevant gist (more in the major subclass) unaltered as well as the relevant gist (the major subclass is part of the superclass), so that the effect is unexplained. Or take the question using three terms (the modified question, “Testing the pragmatic approach” section). Mentioning the minor subclass B’ does not increase the salience of the relation of B in A or decrease the salience of the numerosity of the relation between B and B’.

Finally, the proponents of the fuzzy trace theory seem to misunderstand the linguistic characteristics of the inclusion question. Reyna (1991) examined the linguistic account of the performance and acknowledged the fact that children’s erroneous answers reflect subclass comparison. However she claimed that the interpretation of the question that leads to error is not due to linguistic principles, but rather to a cognitive illusion due to the way the information is presented, which results in the child’s choice of one of the possible interpretations of the question. The question is recognised as ambiguous but this does not create the illusion. This stems from the quantitative information, which is unnecessary to solve the task, and renders the subclass relation salient. In brief, it is because the children attempt to make numerical comparisons that they make subclass comparisons. Reyna put forward three arguments in support of this claim.

First, she claimed that the direction of the developmental data is contrary to the predictions of the psycholinguistic account. This is based on Shipley’s (1979) analysis which considers the exclusive comparisons as ungrammatical and only the inclusive comparisons grammatical; consequently, as the children grow older, they would shift from a grammatical to an ungrammatical interpretation, which is implausible. However, this critique is pointless because it is directed at a hypothesis that is not part of the linguistic pragmatic theory (and is clearly erroneous).

Second, Reyna claimed that the experimental data rule out a causal role for the linguistic factor. This claim is based on two observations. One, mentioned earlier, is that children requested to repeat the question do not substitute the minority hyponym to the hypernym (Brainerd and Kaszor 1974). We have shown (“The subclass-to-subclass comparison” section) that the premises of this argument are unfounded. Two, Brainerd and Kingma (1985) found that numerical probes given after the class inclusion question of the type “How many A were there in the picture, a or b’ ?” (where a and b’ are the numbers of elements in A and B, respectively) were answered correctly, that is, the children did not substitute b’ for a. This is as inconclusive as the previous manipulation because the linguistic theory does not predict that the hypernym in isolation should refer to the minor subclass.

Third, Reyna attributes to the linguistic approach the claim that the inclusive interpretation of the hypernym is the basic one. It follows that older children (and adults) who are more likely to suppose that the experimenter does not ask a question to which she already knows the answer (because the hypernym refers to A, which is the preferred interpretation) would be enclined to choose the alternative, exclusive, interpretation. Consequently one should observe an increase in erroneous responses with age. This argument is flawed for two reasons. One, the claim is wrongly attributed. The correct linguistic approach claims that there is ambiguity and that disambiguation depends on attribution processes, which themselves vary developmentally, so that there is no such a thing as a basic interpretation. Two, the process of attribution is very superficially sketched. It is correct that the older children know that the experimenter knows how to answer whether there are more A than B’, but it should not be forgotten that they also know whether there are more B than B’. The meta-knowledge is necessary but insufficient to suggest an interpretation. The essential point that is missing in this account is what guides the child in his interpretation, namely considerations of relevance. It is because showing mastery of the inclusion relation has become more relevant for the older children than showing mastery of the exclusion relation that they opt for the former. The mature child prefers to show that he knows that there is more in the whole than in the part than to show that, e.g., five is greater than three. To conclude, Reyna (1991) misrepresented the linguistic account and consequently her arguments to refute it are flawed.

In sum, the claim, repeated in Reyna and Brainerd (1995), that wording allows the class-inclusion error but does not create it is clearly incorrect. On the contrary, throughout the present paper it has been shown that the ambiguous formulation of the question is crucial: Whenever a manipulation succeeds in facilitating a correct response it does so by suppressing the ambiguity of the question.

## Conclusions

The essential proposal that has been developed and tested in this paper is that the response to the class inclusion question depend crucially on the “logic of conversation” at work in experimental settings, and more precisely on the child’s interpretation of the question. The question has been submitted to a pragmatic filter. This has been done at two levels of analysis. One, we have performed a fine-grained, or micro-level, analysis and an experimental test of how a question affected by the privative ambiguity of one of its lexical components can receive one interpretation or the other, depending on a variety of factors such as the previous use of the names (hyponym and hypernym) by the interlocutor or their order in the sentence. Second, and more important, we have performed a macro-level analysis of how an ambiguous question uttered by an experimenter (or, to generalise, a teacher) can receive one or the other of these interpretations on the basis of a search for relevance, whereby different children attribute different intentions to the questioner, within the limits of their own metacognitive knowledge. In the end, it is the interaction between these two factors, viz. attribution of intentions and metacognition, that determines the child’s answer and consequently the level of performance inferred by the experimenter.

## Footnotes

1 Before later developments of pragmatic theory a few investigators of child development demonstrated the crucial role of the attribution of intentions by the child to the experimenter. For example, McGarrigle and Donaldson (1975) showed a sharp increase in performance on conservation of length and number when the intervention on the materials was non intentional (a Teddy bear's action) rather than intentional (the experimenter's action). Since this pioneering study, several investigators have developed theoretical and empirical work on the role of participants' and respondents' expectations about experimenter's and inquirer's intentions (Bless et al. 1993; Hilton 1995; Norenzayan and Schwarz 1999; Schwarz 1996) and concerning children (Siegal 1997).

2 Anecdotically, it is not uncommon for children at this age to demonstrate amusement and satisfaction by a smile accompanying their response: You and me know what the smart response is.

3 Anecdotically, but significantly, in a pilot study two children stated that there were no fruit ("there is zero fruit"), as if after they had shown all the fruit (called "pears" and "bananas") it could not be the experimenter's intention to ask again for the same extension, so that "fruit" had no reference. (On the contrary, the hypernym can still refer, namely to B', after only one subclass has been mentioned, as is the case in the standard question).

4 It is noteworthy that in later writings, to explain the superiority of performance with collections over classes, Markman (1989) insisted more on the greater ease to represent collections than classes, based on the notion that the "mutual exclusivity principle" (the child's assumption that category terms are mutually exclusive) is respected for collections (an object *is an* oak but *is part of* a forest) whereas it is not for classes (an object both *is an* oak and *is a* tree).

5 We have shown that the improvement in performance consists in an increase in the frequency of subclass to superclass comparisons and a decrease in subclass to subclass comparisons. In focusing on these two responses and the strategies that lead to them we have not considered a third strategy, namely guessing, which has been shown to be present in a sizeable proportion of the younger children (up to 40 %, but only among 4- to 5- year olds: see Hodkin 1987; Thomas and Horton 1997). It is unlikely that random responding could affect ours results since, by definition, it has an equal contribution to inclusive and non-inclusive responses.

6 Brainerd and Reyna (1995) exploited the task to study childrens' memory and make detailed predictions about the immediate recall of numerical values of the classes before and after answering the class inclusion question. This is beyond the scope of the present paper.
